# CD25 targeting with the afucosylated human IgG1 antibody RG6292 eliminates regulatory T cells and CD25+ blasts in acute myeloid leukemia

**DOI:** 10.3389/fonc.2023.1150149

**Published:** 2023-05-02

**Authors:** Laurène Pousse, Koorosh Korfi, Bruno C. Medeiros, Marco Berrera, Nadine Kumpesa, Jan Eckmann, Idil Karakoc Hutter, Vera Griesser, Vaios Karanikas, Christian Klein, Maria Amann

**Affiliations:** ^1^Roche Pharma Research and Early Development (pRED), Roche Innovation Center Zurich (RICZ), Schlieren, Switzerland; ^2^Genentech, Inc. Hematology Department, South San Francisco, CA, United States; ^3^Roche Pharma Research and Early Development (pRED), Roche Innovation Center Basel (RICB), Basel, Switzerland; ^4^Roche Pharma Research and Early Development (pRED), Roche Innovation Center Münich (RICM), Penzberg, Germany

**Keywords:** acute myeloid leukemia (AML), leukemic stem cells (LSC), regulatory T cell (Treg), CD25, antibody dependent cellular cytotoxicity (ADCC)

## Abstract

**Background:**

Acute Myeloid leukemia is a heterogeneous disease that requires novel targeted treatment options tailored to the patients’ specific microenvironment and blast phenotype.

**Methods:**

We characterized bone marrow and/or blood samples of 37 AML patients and healthy donors by high dimensional flow cytometry and RNA sequencing using computational analysis. In addition, we performed ex vivo ADCC assays using allogeneic NK cells isolated from healthy donors and AML patient material to test the cytotoxic potential of CD25 Mab (also referred to as RG6292 and RO7296682) or isotype control antibody on regulatory T cells and CD25+ AML cells.

**Results:**

Bone marrow composition, in particular the abundance of regulatory T cells and CD25 expressing AML cells, correlated strongly with that of the blood in patients with time-matched samples. In addition, we observed a strong enrichment in the prevalence of CD25 expressing AML cells in patients bearing a FLT3-ITD mutation or treated with a hypomethylating agent in combination with venetoclax. We adopted a patient-centric approach to study AML clusters with CD25 expression and found it most highly expressed on immature phenotypes. Ex vivo treatment of primary AML patient samples with CD25 Mab, a human CD25 specific glycoengineered IgG1 antibody led to the specific killing of two different cell types, CD25+ AML cells and regulatory T cells, by allogeneic Natural Killer cells.

**Conclusion:**

The in-depth characterization of patient samples by proteomic and genomic analyses supported the identification of a patient population that may benefit most by harnessing CD25 Mab’s dual mode of action. In this pre-selected patient population, CD25 Mab could lead to the specific depletion of regulatory T cells, in addition to leukemic stem cells and progenitor-like AML cells that are responsible for disease progression or relapse.

## Introduction

Acute Myeloid Leukemia (AML) is characterized by the aberrant proliferation of immature myeloid cells that originate in the bone marrow (BM). AML is a difficult to treat disease for which cure is mostly limited to younger patients eligible for intensive chemotherapy and allogeneic stem cell transplantation (allo-SCT). However, the combination of a hypomethylating agent (HMA) and BCL-2 inhibitor venetoclax (VEN) greatly improved the response rates and survival in older patients (1, 2).

In recent years, leukemic stem cells (LSCs) have been identified as the cell population that propagates the leukemic pool, resists to conventional therapies, and drive disease relapse; therefore, targeting and eliminating this stem cell pool is considered as the ultimate cure for AML (3, 4). Recent studies have identified cellular markers that distinguish LSCs from normal hematopoietic stem cells, which enable selective targeting of these malignant cells while sparing their normal counterparts. CD25, also known as the α-subunit of the interleukin-2 receptor (IL2RA), is a cell surface molecule that is abnormally expressed on LSCs in a subset of AML patients (5–7) and its expression is associated with lower survival (8). More conventionally however, CD25 is constitutively expressed on regulatory T cells (Tregs) and allows binding of IL-2 with high affinity to the ternary IL-2Rαβγ complex, thereby enabling Tregs to act as IL-2 sink and deprive other immune cells of this important cytokine (9). The frequency of these immunosuppressive Tregs is shown to be higher in AML bone marrow than healthy bone marrow and their abundance correlates with poorer outcome in AML patients (10–12). Additionally, the ratio of intratumoral Tregs to effector T cells is also known to be predictive of clinical outcome in several solid tumors in humans (13, 14). Therefore, targeting CD25 provides not only an opportunity to eliminate the LSCs but also creates an immune permissive bone marrow microenvironment by eliminating Tregs.

We had previously reported CD25 Mab, an afucosylated, IL-2 non-blocking human IgG1 antibody, that was shown to efficiently deplete Tregs in humans and solid tumor bearing mice (15, 16), while allowing binding of IL-2 to effector T cells and the formation of anti-tumor adaptive immune responses (17). CD25 Mab binds to CD25+ target cells and its crystallizable fragment (Fc) to Fc receptors (FcR) expressed on the surface of effector cells, such as FcgRIIIa on Natural Killer (NK) cells, monocytes and macrophages. It mediates killing of target cells through antibody-dependent cellular cytotoxicity (ADCC) and antibody-dependent cellular phagocytosis (ADCP). CD25 Mab is currently being investigated in phase I monotherapy study (NCT04158583) as well as in phase Ib clinical trial in combination with the PD-L1 antibody atezolizumab (NCT04642365) in solid tumor indications and has not yet been clinically evaluated in leukemia.

In this study, we perform an in-depth characterization of immune composition of AML blood and bone marrow and the phenotype of CD25+ blasts, coupled to molecular profiling of the disease. We also investigate CD25 Mab’s potential dual mode of action through depleting suppressive Tregs and eliminating CD25+ AML cells. This study may serve as basis for the identification of a patient population for which CD25 Mab could be a promising therapeutic option.

## Materials and methods

### Primary human samples

Human peripheral blood mononuclear cells (PBMC) and bone marrow mononuclear cells (BMMC) of AML patients were purchased from Discovery Life Sciences (Huntsville, Ala, USA) and ProteoGenex (Los Angeles, CA, USA). Briefly, diseased BMMC were processed from bone marrow aspirates and healthy BMMC from bone marrow reaming obtained during hip replacement surgery. Frozen healthy BMMC were purchased from Discovery Life Sciences. All human samples were collected under the approval of the relevant Institutional Review Board (IRB) or Ethics Committee. Patient samples metadata are available in **Supplementary Tables S1**, **S2**; Patient IDs were anonymized. All AML patients and healthy BM donors provided written informed consent. All primary human samples except NK cells were cryopreserved. For NK cell isolation healthy donor (HD) PBMC were isolated freshly from buffy coats (Zurich Blood Donation Center, Switzerland) using standard density-gradient centrifugation.

### Cell lines and antibodies

Pfeiffer tumor cells (ATCC, Manassas, VA, USA), an established large B-cell lymphoma suspension cell line, were cultured in RPMI 1640 medium (Gibco) containing 20% FBS (Gibco), 1X Glutamax (Gibco), 1X Non Essential Amino Acids (NEAA, Gibco) and 1% Sodium Pyruvate (Gibco). EOL-1 tumor cells (DSMZ, Braunschweig, Germany), an established acute myeloid leukemia suspension cell line, were cultured in RPMI 1640 medium (Gibco) containing 10% FBS (Gibco), 1X Glutamax (Gibco), 1X NEAA (Gibco) and 1% Sodium Pyruvate (Gibco).

AML22 cells originated from an AML patient and were expanded by implantation in immunodeficient NSG mice. AML22 cells were thawed and used directly on the day of the ADCC assay.

CD25 Mab, also referred to as RG6292 is an afucosylated human IgG1 mAb produced as previously described using the GlymaxX technology that confers an enhanced ADCC capacity of CD25 expressing target cells (17). Ultra-LEAF purified human IgG1 isotype control antibody was purchased from Biolegend, San Diego, CA, USA (QA16A12).

### RNA sequencing

RNA and DNA were simultaneously isolated from frozen cells using the Qiagen AllPrep DNA/RNA Mini Kit (QIAGEN^®^, Venlo, The Netherlands) according to the manufacturer’s instructions. Residual genomic DNA was removed during the extraction using the RNase-free DNase set (QIAGEN^®^). RNA was quantified using the Nanodrop 2000 spectrophotometer and quality was assessed on a Bioanalyzer using RNA 6000 Nano chips (Agilent Technologies^®^, Santa Clara, CA, USA). RNA sequencing libraries were prepared using the Illumina TruSeq Stranded mRNA kit according to the manufacturer’s procedure. Libraries were sequenced on a NovaSeq 6000 instrument (Illumina Inc. San Diego, CA, USA) at a targeted read depth of 25M paired-end reads per sample. RNASeq paired-end reads were mapped onto the human genome (build GRCh38) with read aligner STAR version 2.7.3a using default mapping parameters (18). Gene reads counts were calculated using the featureCounts software (19) and normalized as transcripts per million (tpm). The RNA sequencing datasets generated during the current study are available at the NCBI Sequence Read Archive (SRA) under the accession ID PRJNA855458 and PRJNA855476 for Study #1 and #2, respectively.

### FLT3-ITD analysis

FLT3-ITD analysis was performed at MLL Dx GmbH (Münich, Germany). Briefly, internal tandem duplications (ITDs in the fms-like tyrosine kinase 3 (FLT3) gene were detected by DNA fragment analysis with a sensitivity of 1%. FLT3-ITD allelic ratio (AR) was calculated as the ratio of the area under the curve of mutant to wild-type alleles.

### Quantification of CD25 density by flow cytometry

BD quantibrite™ Beads (BD Biosciences, San Jose, CA, USA) were used to estimate antibodies bound per cell (ABC) which is equivalent to the number of PE molecules per cell if PE:mAb ratio is 1:1. In the case of monovalent binding of the PE-conjugated anti-human CD25 antibody (clone 24212, R&D Systems), the number of CD25 molecules would be equivalent to the number of PE molecules on the cell surface (20).

Cellular assay samples and beads were acquired with the same instrument settings on a 5-laser Symphony instrument (BD). Linear regression of Log10 PE molecules per bead against Log10 fluorescence as well as interpolation of number of PE molecules per cell were determined according to the manufacturer’s instructions. Antibody density was quantified on CD25 expressing target cells (Pfeiffer, EOL-1, AML22) used in the *in vitro* ADCC assay.

### Flow cytometry panel design, staining and acquisition

We developed flow cytometry panels of 26 or 39 cellular markers to characterize samples in Study #1 and #2, respectively. We followed recent guidelines (21) for panel design and optimal validation. Of note, all antibodies were titrated and a combination of Fluorescence-minus-one (FMO) controls as well as biological controls (cell populations lacking one or a set of markers) were utilized to validate the panels.

Cryopreserved AML patient and HD samples were thawed in DMEM/F-12 medium (Gibco) containing 10% FBS (Gibco). Cells were incubated with Human TruStain FcX (Biolegend) and stained with FSV440UV (BD) or Zombie NIR (Biolegend) viability dye diluted in PBS. Then, cells were stained with fluorochrome-conjugated antibodies against surface antigens (listed in **Supplementary Tables S3**, **S4**) in staining buffer containing FACS buffer (PBS containing 0.5% BSA) and Brilliant Stain buffer (BD). Cells were fixed and permeabilized using FoxP3 Transcription Factor Staining Set (eBioscience, San Diego, CA, USA) and subsequently stained against intra-cellular antigens (listed in **Supplementary Tables S3**, **S4**) in 1X PERM buffer. Samples were acquired with a 5-laser A5 Symphony instrument (BD) for study #1 and ADCC assay with cell lines. Patient samples in study #2 and ADCC assay with patient material were acquired with a 5-laser Aurora spectral cytometer (Cytek, CA, USA).

Pre-processing steps (applying of compensation matrix, gating on live single cells) were performed with FlowJo v10.6.2 (Ashland, OR, USA) or SpectroFlo^®^ software.

For samples acquired with the Aurora spectral cytometer, unmixed files were checked individually and manual spillover compensation adjusted when necessary using the integrated software. Of note, only minor modifications were needed. Events of FCS files that passed all quality control steps were exported for further computational analysis with R.

### Computational flow cytometry data analysis

The main steps of the computational analysis workflow are summarized in **Supplementary Figure S1**. FCS files were loaded into R and processed as described in the vignette of the flowCore R package. Logicle transformation was applied onto the expression matrix. Density plots for each marker were visually inspected and thresholds defined to allow for the quantification of positive and negative expression, similarly to positivity cutoffs assigned by traditional manual gating.

Dimensionality reduction was performed using the uniform manifold approximation and projection (UMAP) algorithm as part of the *uwot* R package. Unsupervised clustering was performed in the high-dimensional space with PhenoGraph (22) and results thereof visualized on the UMAP plot as a color overlay. Algorithm-generated clusters were merged and annotated manually based on marker expression in order to arrive at biologically meaningful cell populations. Downstream analysis reported cell abundance, marker expression as well as fractions of positive cells where appropriate.

This workflow was applied to PBMC and BMMC samples separately. Lymphoid populations were analyzed in this first iteration step. Non-lymphoid cells were then subjected to a second round of dimensionality reduction and clustering to gain a better resolution of the myeloid clusters. Cells negative for CD45 expression and all other markers present in the panel (non-immune cells) as well as very small clusters (< 0.05%) were filtered out. Cell populations with less than 50 event counts recorded were excluded from the analysis. Myeloid cell abundance and clusters presented in the figures refer to non-lymphoid cells after exclusion of non-immune cells and very rare clusters according to the aforementioned filters. For the detailed analysis of the CD25+ AML clusters, we used a patient-centric approach and applied the computational analysis workflow described above for each patient separately.

CD25+ cell populations identified by computational analysis were validated by manual gating (see **Supplementary Figure S2** for a representative example of the gating strategy) and Median Fluorescence Intensity (MFI) values exported. Figures were generated using the ggplot2 package in R 4.0.1 or with Prism v8.4.2 (GraphPad Software, San Diego, CA, USA).

### NK cell isolation

NK cells were isolated from HD PBMC using the human NK cell isolation kit (Miltenyi Biotec, Bergisch Gladbach, Germany) and activated overnight in RPMI 1640 medium (Gibco) containing 10% FCS (Gibco), 1X Glutamax (Gibco) and 100 U/mL Proleukin/Aldesleukin (Novartis, Basel, Switzerland).

### ADCC assay

CD25 expressing target cells were mixed with activated primary NK cells at a 2 to 1 effector to target cell ratio (80,000 NK cells and 40,000 target cells per well) in assay medium (RPMI 1640 (Gibco) containing 2% FBS (Gibco) and 1X Glutamax (Gibco)). Total cell counts of patient samples (n=4) were adjusted to obtain 40,000 CD25+ AML cells per well and number of NK cells was kept constant. In the same experiment, Treg depletion was assessed for three out of four patients with sufficient Treg content in the untreated patient sample (even count > 50). Tregs represent on average 1% of viable BMMC. In the ADCC assay with HD BM, 400,000 unsorted BMMC were added per well to obtain a 20:1 NK to Treg ratio. Compounds (CD25 Mab or isotype control) were added to U bottom 96-well plate (TPP) at a 7-fold dilution series, with a starting concentration of 21 µg/mL. Assay plates were placed on an orbital shaker for 5 minutes at 300rpm to mix cells and antibodies. After 15 minutes of pre-incubation at room temperature, samples were incubated at 37°C/5%CO_2_ for 17-20 hours. In the ADCC assay with cell lines, killing activity was calculated based on the target cell event count normalized to the number of target cells in the absence of allogeneic effector NK cells and compounds. In the cytotoxicity assay with patient material, Precision Count BeadsTM (Biolegend) were added before sample acquisition and killing activity calculated based on absolute counts (number of cells/µl). Normalized cell counts were selected as cytotoxicity readout in order to specifically identify different cell populations within the complex composition of patient samples by staining ADCC samples with the same flow cytometric panel used for baseline characterization.

### Cytokine detection

For the ADCC assay with patient material, cell-free supernatants were collected at endpoint (20 hours) and stored at -80°C. Samples were thawed and cytokine content analyzed by Luminex technology using Bio-Plex Pro Human Cytokine 27-plex and IL2RA single-plex assays (Bio-Rad, Hercules, CA, USA) according to the manufacturer’s instructions. Sample acquisition and cytokine concentrations were determined using a Bio-Plex 200 System (Bio-Rad) and its integrated software. Figures were generated with Spotfire v11 (TIBCO Software, Palo Alto, CA, USA) or Prism v8.4.2 (GraphPad Software).

### Statistical analysis

Statistical significance was evaluated using Prism v8.4.2. Statistical tests are described in the Figure legends. Significance levels are indicated as follows: ns or not indicated, not significant, *P* > 0.05; *, *P* ≤ 0.05; **, *P* ≤ 0.01; ***, *P* ≤ 0.001; ****, *P* ≤ 0.0001.

## Results

### Bone marrow composition of AML patients is altered compared to healthy donors

**Figure 1:**
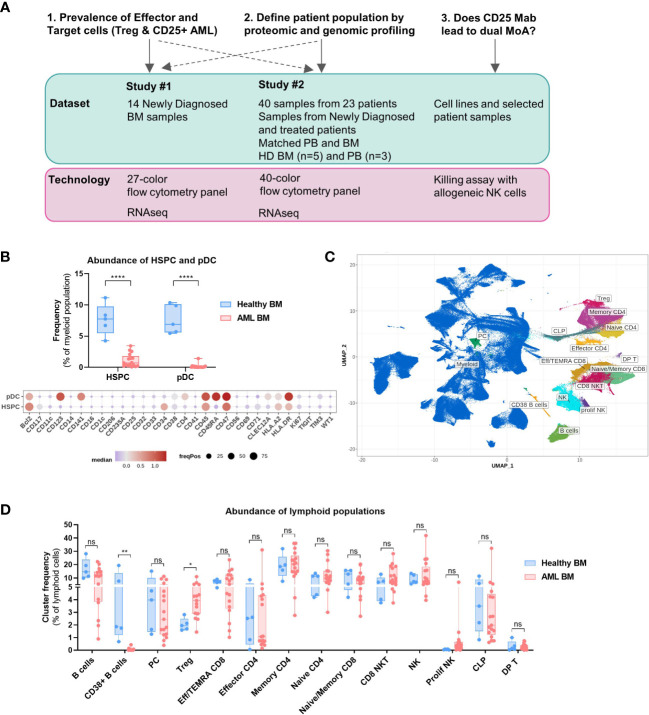
Bone marrow composition differs between healthy donors and AML patients. **(A)** Overview of the study. Shown are the key questions with associated datasets and technologies used. **(B–D)** Data obtained with BM samples in Study #2. **(B)** Top: Abundance of pDC and HSPC in the bone marrow of healthy donors (n=5) and AML patients (n=17). Multiple t tests, Holm-Sidak correction for multiple comparisons. Bottom: Dot plot depicting the expression of 30 selected markers on HSPC and pDC identified in combined clustering of healthy and AML bone marrow samples. Color code indicates the median expression (logicle transformed MFI); point size indicates the fraction of cells positive for each marker. **(C)** Lymphoid clusters overlaid on UMAP embedding of all BM samples (healthy and AML). **(D)** Differential abundance of all lymphoid clusters between Healthy and AML BM as a proportion of lymphoid cells. Multiple t tests, not corrected for multiple comparisons. BM, Bone marrow; PB, Peripheral blood; HD, Healthy donor; Treg, Regulatory T cell; MoA, Mode of action; HSPC, Hematopoietic Stem and Progenitor cell; pDC, Plasmacytoid dendritic cell. Significance levels are indicated as follows: ns or not indicated, not significant, P > 0.05; *P ≤ 0.05; **P ≤ 0.01; ***P ≤ 0.001; ****P ≤ 0.0001.

We performed a detailed characterization of AML BM samples by high dimensional flow cytometry analysis. Samples from 14 newly diagnosed patients were analyzed in Study #1 whereas Study #2 contained samples from patients at diagnosis or treated with different treatment categories (chemotherapy, HMA or HMA-VEN). We investigated the cellular composition of HD BMMC (n=5) compared to that of AML patients (n=17 samples, median age of 63) using a 40-color flow cytometry panel (**Supplementary Table S4**) in order to identify all major cell types and conducted a detailed phenotypic analysis of the AML blasts and T cells (**Figure 1A**, Study #2).

We first studied the prevalence of healthy non-lymphoid populations to identify significant alterations in the composition of AML BM compared to that of healthy volunteers. We used the absence or low expression of CD45RA as a marker to discriminate normal hematopoietic stem and progenitor cells (HSPCs) from leukemic stem and progenitor cells within the CD34+CD38+/- population (23, 24). We observed a significantly lower frequency of HSPCs in AML BM (**Figure 1B**), likely due to the aberrant proliferation of malignant myeloid progenitors. Importantly, CD25 expression was absent on HSPCs (**Figure 1B**); therefore, the risk of depleting normal hematopoietic stem cells by targeting CD25 is low. The frequency of plasmacytoid dendritic cells (pDCs) was also decreased.

We also carried out a detailed prevalence analysis of the lymphoid populations and annotated clusters are displayed on the UMAP plot (**Figure 1C**), while myeloid clusters are shown in **Supplementary Figure S3B**. The abundance of CD38+ B cell clusters was lower in the BM of AML patients and interestingly, there was a higher proportion of Tregs as compared to HD BM (**Figure 1D**, Tregs = 2% of lymphoid cells in HD vs 4.3% in AML). We observed a high degree of heterogeneity in the abundance of the other conventional T cell subsets in AML patients and no statistically significant differences with HD BM.

### CD25 is expressed on BM Tregs of AML patients

**Figure 2:**
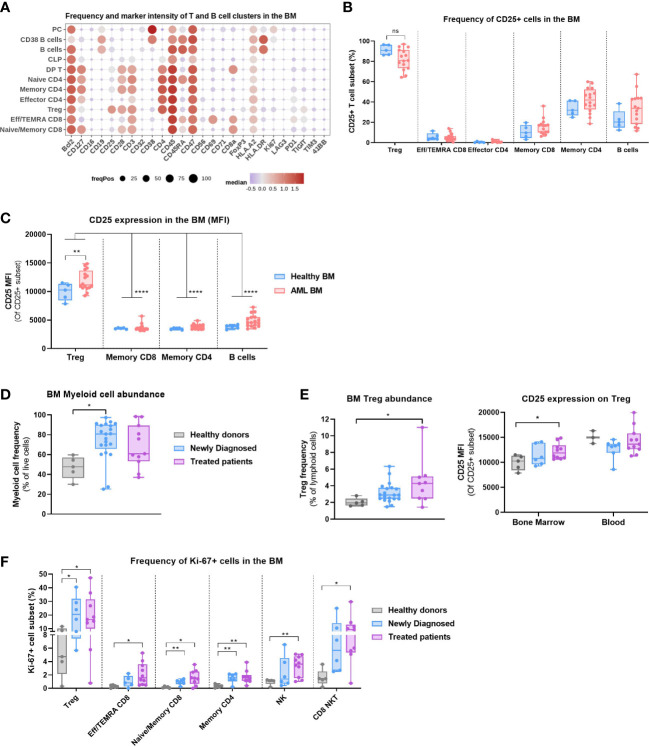
Regulatory T cells are present in the bone marrow of AML patients and express CD25. **(A–C)** Data obtained with BM samples in Study #2. **(A)** Dot plot depicting the expression of 26 markers on annotated T and B cell clusters. Color code indicates the median expression (logicle transformed MFI); point size indicates the fraction of cells positive for each marker. **(B)** Frequency of CD25+ mature T and B cell subsets. Two-way ANOVA, Sidak’s multiple comparison test. **(C)** CD25 expression (MFI) on selected T and B cell subsets. Two-way ANOVA, Sidak’s multiple comparison test (for the comparison of Healthy vs AML) or Tukey’s multiple comparison test (for the comparison of expression between cell subsets). **(D)** Myeloid cell abundance in the bone marrow of HD (n=5), Newly Diagnosed (n=22) and Treated patients (n=11). Data compiled from Study #1 and #2. Kruskal-Wallis with Dunn’s multiple comparison test. **(E)** Left: Treg abundance in the bone marrow of HD, Newly Diagnosed and Treated patients. Data compiled from Study #1 and #2. Kruskal-Wallis with Dunn’s multiple comparison test. Right: CD25 expression on Treg in the blood vs bone marrow of HD, Newly Diagnosed and Treated patients in Study #2. Multiple t tests, Holm-Sidak correction for multiple comparisons. **(F)** Frequency of Ki67+ cell populations between HD (n=5), Newly Diagnosed (n=7) and Treated patients (n=10) in Study #2. Multiple t tests, not corrected for multiple comparisons. Significance levels are defined in the methods section. P values > 0.05 are not indicated on the graphs. BM, Bone marrow; HD, Healthy donor; Treg, Regulatory T cell; NK, Natural Killer cell. Significance levels are indicated as follows: ns or not indicated, not significant, P > 0.05; *P ≤ 0.05; **P ≤ 0.01; ***P ≤ 0.001; ****P ≤ 0.0001.

We then studied the phenotype of annotated T and B cell clusters identified in the bone marrow (**Figure 2A**) and found that CD25 was expressed on most BM Tregs, whereas memory CD4 T cells and B cells displayed lower frequencies. There were no significant differences in the frequency of CD25+ T and B cell clusters between HD and AML BMs (**Figure 2B**). We also studied CD25 expression level (MFI) on lymphoid clusters with significant CD25 expression (**Figure 2C**) and concluded that CD25 expression on Tregs was higher than on Memory CD4 and CD8 T cells as well as B cells, corroborating expression patterns observed in solid tumors (25).

Next, we evaluated the impact of prior treatment on immune cell composition of AML BM. We observed a higher myeloid cell content in the newly diagnosed patients, a surrogate measure of the disease burden (**Figure 2D**). Furthermore, the abundance of Tregs within the lymphoid compartment was higher in the bone marrow of treated patients than in HD (**Figure 2E**), which indicates that treatment regimen may gradually increase the proportion of these immunosuppressive cells. Moreover, the level of CD25 expression on BM Tregs of treated patients was moderately higher than on HD Tregs, although overall lower than in peripheral blood (**Figure 2E**). We also evaluated the proportion of proliferating T cells and NK cells and observed a significantly higher frequency of Ki-67+ Tregs in AML samples. The frequency of Ki-67+ T and NK cells was generally higher in newly diagnosed patients compared to HD, which may be indicative of an ongoing immune response, and was further increased in patients with prior therapy (**Figure 2F**). Of note, most treated patient samples were collected from relapse/refractory patients (see **Supplementary Table S2**).

The results indicate that proliferating Tregs with an increased CD25 expression and low CD45RA, markers of an activated phenotype (14), are present in the bone marrow of AML patients and may therefore contribute to an immunosuppressive microenvironment.

### Peripheral blood offers a window into the bone marrow composition

**Figure 3:**
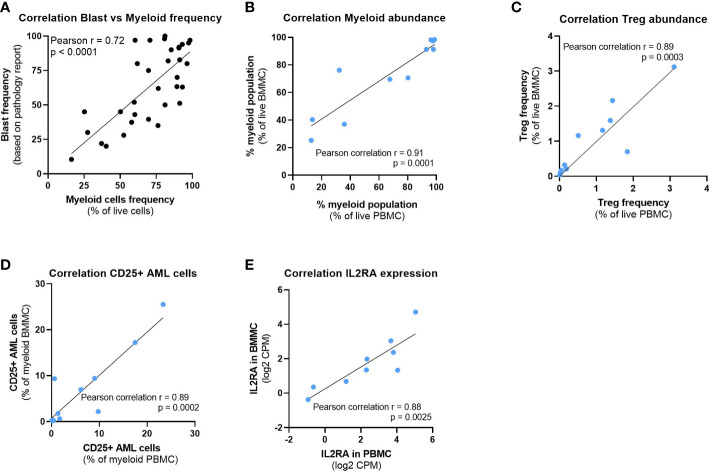
Peripheral blood offers a window into the bone marrow composition. **(A)** Correlation between the myeloid cells frequency detected by high dimensional flow cytometry analysis and the blast frequency as per pathology report. **(B–D)** Correlation between time matched PBMC and BMMC samples from 11 patients of **(B)** Myeloid cell abundance, **(C)** Treg abundance and **(D)** CD25+ AML cells frequency. **(E)** Correlation of IL2RA gene expression between time matched PBMC and BMMC samples from 9 patients. Pearson correlation r and P values are depicted on the graphs. BMMC: Bone marrow mononuclear cell; PBMC: Peripheral blood mononuclear cell; Treg: Regulatory T cell; CPM: Counts per million.

We sought to study the composition of time-matched blood and bone marrow samples available for 11 patients. We confirmed that the myeloid cell frequency determined by high dimensional flow cytometric analysis correlated strongly with the blast frequency reported by the pathologist (**Figure 3A**). Myeloid cell abundance was therefore used as a surrogate for the disease burden, which was noticeably high in the majority of patients. The abundance of myeloid cells (**Figure 3B**), Tregs (**Figure 3C**) and CD25+ AML cells (**Figure 3D**) was comparable between blood and bone marrow samples in our study. In addition, we performed RNA sequencing of the same patient samples characterized by flow cytometry and observed a similar correlation in the *IL2RA* expression of time matched samples (**Figure 3E**). Therefore, we included blood samples in the phenotype analysis of AML cells.

### Patient-centric phenotype analysis of CD25+ AML clusters

**Figure 4:**
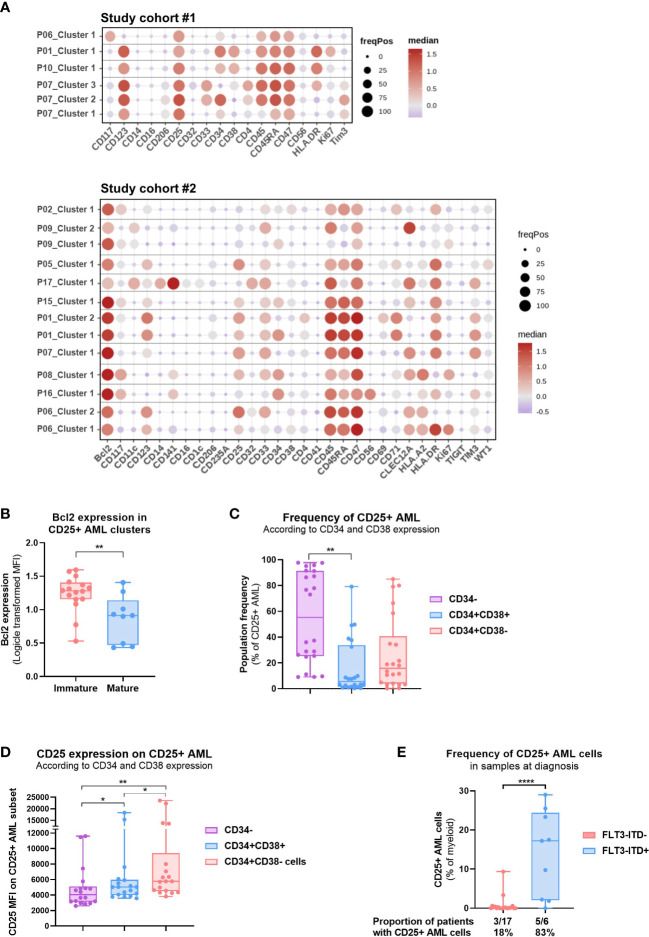
Patient-centric approach allows the phenotypic analysis of CD25+ AML clusters. **(A)** Dot plot depicting the per patient phenotypic analysis of CD25+ AML clusters identified in 14 patients in Study #1 (top) and Study #2 (bottom). See Methods for details. Color code indicates the median expression (logicle transformed MFI); point size indicates the fraction of cells positive for each marker. **(B)** Bcl-2 expression (logicle transformed MFI) in CD25+ AML clusters identified in Study #2. Unpaired t test. **(C)** Frequency of CD25+ AML subsets defined according to CD34 and CD38 expression (CD34-, CD34+CD38+, CD34+CD38-) in the 14 pre-selected patients shown in **(A)** Data compiled from BMMC and PBMC samples analyzed in Study #1 and #2. One-way ANOVA, Tukey’s multiple comparison test. **(D)** CD25 expression (MFI) on CD25+ AML subsets defined according to CD34 and CD38 expression (CD34-, CD34+CD38+, CD34+CD38-). Data compiled from BMMC and PBMC samples analyzed in Study #2. One-way ANOVA, Tukey’s multiple comparison test. **(E)** Abundance of CD25+ AML cells in treatment naïve PBMC or BMMC samples according to the FLT3-ITD status. Data compiled from Study #1 and #2. Unpaired t test. The proportion of patients for which CD25 expression was detected on AML cells is shown below the bar graph. Significance levels are defined in the methods section. P values > 0.05 are not indicated on the graphs. BM: Bone marrow; HSC: Hematopoietic stem cell; Chemo: Chemotherapy; HMA: DNA Hypomethylating Agent; VEN: venetoclax; FLT3-ITD: Internal tandem duplications (ITDs) in the fms-like tyrosine kinase 3 (FLT3) gene. Significance levels are indicated as follows: ns or not indicated, not significant, P > 0.05; *P ≤ 0.05; **P ≤ 0.01; ***P ≤ 0.001; ****P ≤ 0.0001.

We carried out in-depth proteomic and genomic analyses in order to deepen our understanding of the patient population bearing CD25 expression on AML cells. Global clustering analysis conducted by pooling all patients from study #1 revealed significant heterogeneity in the myeloid compartment (**Supplementary Figure S3A**). We subsequently further characterized the BM myeloid compartment by annotating clusters based on the common AML cell surface antigens (e.g. CD123, CD33, CD34), and intracellular marker WT-1 (**Supplementary Figures S3B–D**). Importantly, CD47 was the only marker detected in all the patients and AML clusters revealed diverse phenotypes (**Supplementary Figures S3C, D**) which reflects the underlying heterogeneity in AML. Therefore, to study the AML cell clusters that express CD25, we clustered the AML compartments in a patient-centric manner. We identified four patients in Study #1 and ten patients in Study #2 to express CD25 and evaluated the phenotype of the CD25+ AML clusters (**Figure 4A**, **S4**). Of note, one to four samples per patient were analyzed based on availability of time-matched PB and BM samples or several time points (refer to **Supplementary Tables S1**, **S2** for details). In addition, one to three CD25+ AML clusters were uncovered for each of the 14 patients. Interestingly, CD25+ clusters also commonly expressed CD34, a marker of immature leukemic stem/progenitor cells. Nevertheless, CD25 expression was also observed on mature AML clusters in a smaller subset of AML patients (e.g. CLEC12A+CD33+), albeit negatively correlated with monocytic AML (e.g. CD14 expressed in 1/19 clusters) (**Figure S4**). Importantly, HSCs were identified in a subset of these patients at low frequencies and showed no detectable CD25 expression (data not shown). Furthermore, we also identified a significant association between higher expression of anti-apoptotic protein BCL-2, and the immature CD25+ AML clusters (**Figure 4B**). In addition, we conducted a bivariate analysis of CD34 and CD38 expression by traditional gating for each patient and found that LSCs (CD34+CD38-) represented on average 26% of the CD25+ AML population (**Figure 4C**). We also studied the CD25 expression level in CD34-, CD34+CD38+ and CD34+CD38- subsets with the CD25+ AML compartment and observed that LSCs expressed CD25 at significantly higher levels than more differentiated AML cells (CD34-) (**Figure 4D**).

We complemented the proteomic analysis with known genetic abnormalities in AML, and particularly investigated previously reported association between CD25 expression and FLT3-ITD mutations (5, 7). We observed a strong enrichment of CD25+ AML cells in treatment-naïve patients with FLT3-ITD mutation as compared to FLT3 wild type (**Figure 4E**). Moreover, we also investigated the impact of prior therapy in relapsed/refractory patients, and intriguingly, patients treated with HMA-VEN displayed a higher frequency of CD25+ AML cells compared to treatment-naïve or chemo-treated patients (**Supplementary Figure S5A**). Further paired diagnostic-relapse analysis is required to evaluate the dynamics of CD25+ AML fractions during AML disease evolution and progression in response to certain therapies.

Our results, therefore, suggest that CD25 could be a promising antigen for targeting LSC and immature AML cells alone or in combination with BCL-2 or FLT3 inhibitors in order to prevent relapse and disease progression.

### CD25 Mab depletes Tregs and CD25+ AML cells

**Figure 5:**
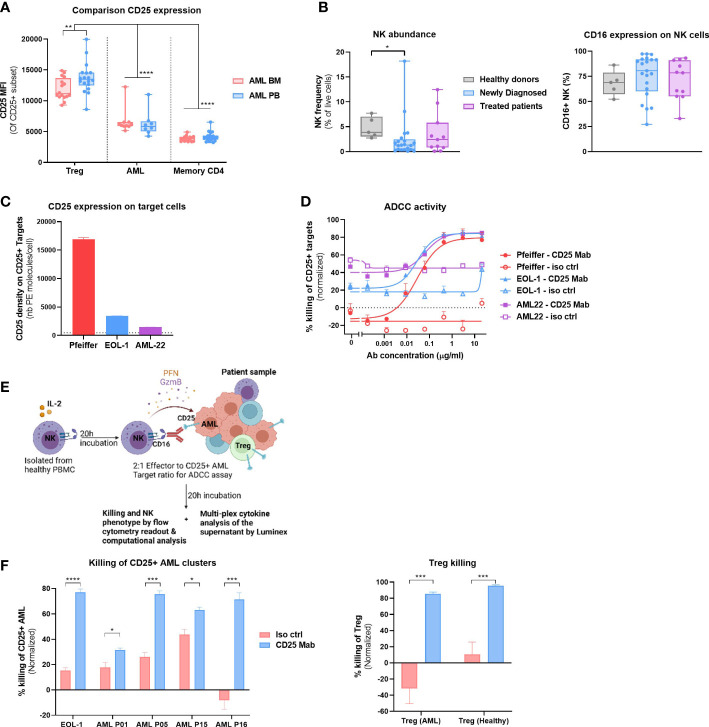
CD25 Mab depletes CD25+ AML and Tregs. **(A)** CD25 expression (MFI) on CD25+ Treg, AML cells or Memory CD4 T cells in Peripheral blood (PB, n = 22) or BM (n = 17) of samples analyzed in Study #2. Two-way ANOVA, Sidak’s multiple comparison test (for the comparison of BM vs PB) or Tukey’s multiple comparison test (for the comparison of expression between cell subsets). **(B)** Left: NK cell abundance (left) and CD16 expression on NK cells (right panel) in the BM of HD (n=5), Newly Diagnosed (n=22) and Treated patients (n=11). Data compiled from Study #1 and #2. Kruskal- Wallis with Dunn’s multiple comparison test. Right: Frequency of CD16+ NK cells. **(C)** Density of CD25 molecules on the surface of live target cells (Pfeiffer, EOL-1 and AML-22) in the absence of effector cells and test compounds was determined using BD quantibrite™ Beads, 17h after ADCC assay initiation. The limit of detection (L.O.D) is represented by the dotted line and corresponds to the lowest number of PE molecules present on the BD quantibrite™ Beads. **(D)** Dose response ADCC activity of the tested compounds (CD25 Mab or isotype control antibodies) as a frequency of CD25+ Target cell killing. Flow cytometric analysis was performed 17h after onset of ADCC assay. Mean +/- SEM represent results obtained with two NK cell donors and technical duplicates. EC50 values were calculated using GraphPad Prism and its inbuilt non-linear regression curve fit (log (agonist) vs response, variable slope, 4 parameter)). **(E)** Graphical representation of the cytotoxicity assay with allogeneic NK cells and patient material to evaluate CD25 Mab dual mode of action hypothesis. Created with BioRender.com **(F)** Left: Killing activity of the tested compounds at 10μg/ml (CD25 Mab or isotype control antibodies) as a frequency of CD25+ AML cell killing. See methods section for a detailed description of the experimental setup. Positive control EOL-1 cell line and four AML patient samples from study #2 were used as target cells. Flow cytometric analysis was performed 20h after onset of ADCC assay. Mean +/- SEM represent results obtained with two NK cell donors and technical duplicates. Right: Treg killing activity as described in left panel. Mean +/- SEM represent results obtained with AML Treg (n=3) or healthy BM Treg (n=2). Multiple t tests, Holm-Sidak correction for multiple comparisons. Significance levels are defined in the methods section. P values > 0.05 are not indicated on the graphs. BM, Bone marrow; Treg, Regulatory T cell; NK, Natural Killer cell; ADCC, Antibody dependent cellular cytotoxicity. Significance levels are indicated as follows: ns or not indicated, not significant, P > 0.05; *P ≤ 0.05; **P ≤ 0.01; ***P ≤ 0.001; ****P ≤ 0.0001.

CD25 Mab preferentially depletes cells with a high CD25 surface density such as Tregs and spares T cells with low CD25 density in preclinical cancer models (17). Therefore, we assessed the CD25 expression level on CD25+ AML cells. We observed CD25 expressed at an intermediate level (**Figure 5A**), as compared to Tregs (high expression) and memory CD4 T cells (low expression). Of note, there was no significant difference in expression neither between PB and BM (**Figure 5A**), nor according to the treatment category (**Supplementary Figure S5B**).

Next, we evaluated our dual mode of action hypothesis, whereby CD25 Mab treatment would lead to a direct cytotoxicity of CD25+ AML cells and depletion of immunosuppressive Tregs through FcR bearing effector cells, such as FcgRIIIa (CD16) expressing NK cells. We evaluated the NK cell abundance in all patient samples in Study #1 and #2 and detected NK cells in most patients (**Figure 5B**, left). Moreover, NK cells of AML patients expressed the ADCC-mediating receptor CD16 at high frequencies, similarly to that of HD, without any noticeable difference between newly diagnosed and treated patients (**Figure 5B**, right).

To assess the capacity of CD25 Mab to eliminate AML cells, we performed an ADCC assay with allogeneic HD NK cells, comparing target cells with a range of CD25 expression levels.

As shown in **Figure 5C**, Pfeiffer cell line expresses relatively high levels of CD25, whereas AML cell line EOL-1 and patient derived AML22 cells harbor a lower number of CD25 receptors on their surface.

Despite the fact that CD25 Mab features an avidity driven binding to CD25 and as a consequence preferentially triggers the killing of cells with high levels of CD25 expression (17), the ADCC capacity of CD25 Mab with EOL-1 and AML-22 as target cells were comparable to that of CD25 high target cells (**Figure 5B**). More than 80% of the target cells were killed by NK cells after 17 hours of co-incubation with CD25 Mab whereas no specific killing was observed with the isotype control antibody. Having demonstrated cytotoxicity of CD25 Mab on EOL-1 cell line and AML22 cells, we also assessed its functional activity with patient material. For this purpose, we selected four patients with the highest frequency of CD25+ AML cells and performed an *ex vivo* ADCC assay (**Figure 5E**). We showed specific killing of CD25+ AML cells in all samples at saturating antibody concentration (**Figure 5F**, left). Moreover, we assessed Treg killing in the same experiment and found that both AML and HD Tregs were efficiently depleted by CD25 Mab (**Figure 5F**, right). Taken together, these results provide a proof of concept of CD25 Mab’s dual mode of action in AML patient samples using HD NK cells.

### NK cell activation phenotype does not correlate with their cytotoxic potential

**Figure 6:**
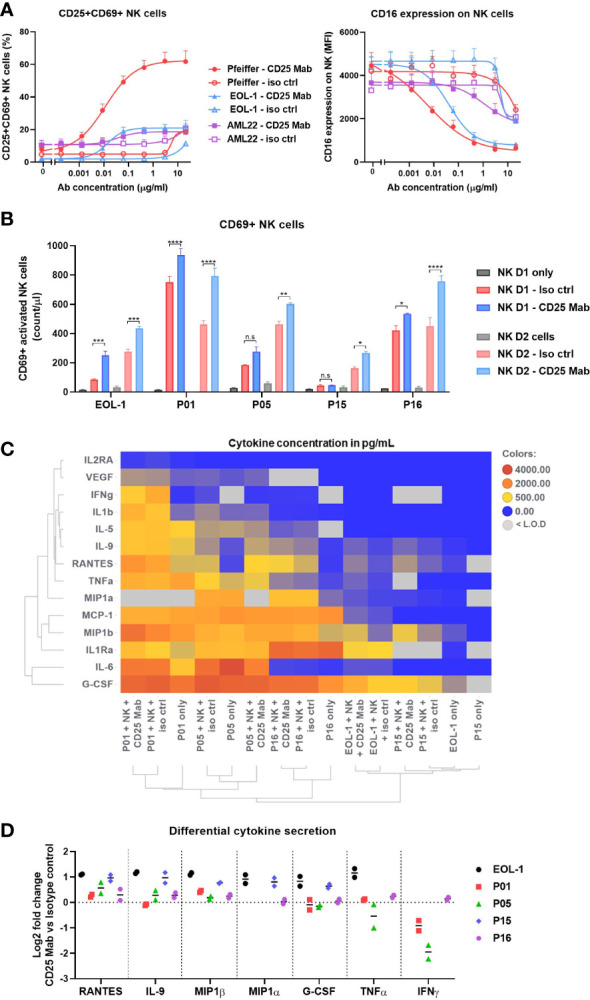
Depth of NK cell activation does not correlate with their cytotoxic activity. **(A)** Abundance of CD25+CD69+ NK cells (left) or CD16 expression (MFI) on NK cells (right) after 16-20h incubation in ADCC assay with Target cells (Pfeiffer, EOL-1 or AML22) treated with CD25 Mab or isotype control antibody. **(B-D)** Data obtained at ADCC assay endpoint (20h). Experimental procedure is described in the **Figure 5E**. **(B)** Abundance of CD69+ NK cells represented as count per µl in the absence or presence of target cells and test molecules. Bar graph represents mean +/- SEM for each NK cell donor with technical duplicates. Two-way ANOVA, Tukey’s multiple comparison test. **(C)** Heatmap of selected cytokine concentrations (mean values in pg/mL) detected in the supernatant by Luminex analysis. Row and column dendrograms represent hierarchical clustering using UPGMA method and Euclidean distance. **(D)** Log2 fold change of cytokine concentration obtained with CD25 Mab treatment as compared to isotype control antibody for seven differentially expressed cytokines. Each pictograph represents the mean of technical duplicates for each NK cell donor. Significance levels are defined in the methods section. P values > 0.05 are not indicated on the graphs. NK: Natural Killer cell; ADCC: Antibody dependent cellular cytotoxicity. Significance levels are indicated as follows: ns or not indicated, not significant, P > 0.05; *P ≤ 0.05; **P ≤ 0.01; ***P ≤ 0.001; ****P ≤ 0.0001.

We sought to examine if target cells with different CD25 expression levels would have an impact on the strength of NK cell activation*. In vitro* ADCC assays showed a minor increase in EC50 with CD25low AML22 target cells as compared to target cells that harbor higher CD25 density, even though the maximum cytotoxic capacity was comparable (**Figures 5C, D**). Additionally, the functionality of NK cells stimulated by CD16 engagement can be measured by the expression of activating receptors such as CD69 and CD25. We observed a strong upregulation of CD25+CD69+ NK cells in a CD25 Mab dose-dependent manner after incubation with CD25high target cells (**Figure 6A**, left).

Moreover, engagement with low, medium or high CD25 density target cells resulted in a modest (EC50 = 1.164µg/ml), intermediate (EC50 = 0.041µg/ml) or strong (EC50 = 0.006µg/ml) down-regulation of CD16 on NK cells, respectively (**Figure 6A**, right). Therefore, the results indicate that the strength of CD16 down-regulation correlates with the CD25 density on the target cells. In the killing assay with patient material, the number of activated CD69+ NK cells was significantly increased after CD25 Mab treatment for all patient samples and the two NK cell donors tested (**Figure 6B**). The highest number of CD69+ NK cells was found with AML P01 patient material, however it was also the smallest specific killing (**Figure 5F**). Therefore, these results suggest that the NK cell phenotype does not correlate with their cytotoxic activity. Considering the high level of heterogeneity in the composition of the patient samples, we sought to analyze the cytokine content in the supernatant at ADCC assay endpoint (20h). Using a multi-plex Luminex assay, we analyzed the 14 cytokines whose concentration was within the assay detection range for at least two AML patient samples (**Figure 6C**). We found relatively high concentrations of pro-inflammatory cytokines in the AML P01, P05 and P16 patient samples, whereas AML P15 and EOL-1 cell line displayed lower cytokine secretion, which demonstrates that the target cells are the main drivers of the cytokine content. In order to investigate if the treatment condition influenced the cytokine secretion by NK cells, we evaluated the Log2 fold change in cytokine concentration of CD25 Mab vs isotype control antibody. We noticed an increase in RANTES, IL-9, MIP1α and MIP1β secretion specifically induced by CD25 Mab for most patient samples (**Figure 6D**). The strongest fold change due to CD25 Mab treatment was observed with EOL-1 cell line and AML P15 sample that had lower baseline cytokine content. In summary, NK cell activating phenotype and ability to secrete pro-inflammatory cytokines seems to be influenced by the CD25 expression on target cells. CD25 Mab treatment specifically increased the frequency of CD69+ NK cells and additional samples are required to study patterns linking activating phenotype and cytotoxic potential.

## Discussion

AML stem cells represent one of the most characterized cancer stem cell populations and their role in disease perpetuation, resistance and relapse has been elucidated over the years. However, novel targeted therapies have not been very successful in eliminating this immature population due to the significant disease heterogeneity and various tumor cell intrinsic and extrinsic factors conferring stemness and protection from cell death (26–28). Immunotherapy is a promising approach for the treatment of AML; however, the lack of universal surface antigen or combination of antigens on AML blasts and LSCs (29) remains a challenge. Here, we showed through in-depth proteomic analysis that CD25 expression is preferentially associated with AML LSC/progenitor population in a subset of patients and is absent from normal HSCs. This has also been recently described by single cell RNA sequencing (30) and targeted surface protein expression analysis (31). However, our understanding of the CD25’s biological importance in LSC maintenance and differentiation is scant and future work with long-term co-culture or colony forming unit assays may shed some light on the functional stem like potential of CD25+ AML cells, and the factors influencing its fluctuating expression (6).

Using ex vivo killing assays with patient material, we showed that allogeneic NK cells elicited potent killing of Tregs and CD25+ AML cells in patient samples incubated with CD25 Mab. In addition to the direct effect on CD25+ AML blasts, depleting Tregs in AML could contribute to the activation of CD8 T cells through re-distribution of IL-2 and therefore creating a more immune permissive microenvironment (17). In contrast, the IL-2 blocking antibodies such as Daclizumab (32) and Basilixumab (33) used in the clinics as immunosuppressive agents, showed inferior anti-tumor efficacy as compared to non IL-2 blocking RG6292 in preclinical models (17). AML patients with monosomy 7 (chr7/7q loss) present with an immunosuppressive BM microenvironment enriched in Tregs and are associated with inferior outcomes (34). Although the CD25 expression on AML blasts in these patients remain to be evaluated, they may benefit from Treg depletion by CD25 Mab. Furthermore, there is evidence that Tregs directly regulate the stemness of AML cells through the release of IL-10 and a PI3K/AKT signaling pathway in preclinical leukemia models (35). There is some evidence that PD-L1 expression by AML cells may directly drive the expansion of PD-1+ Tregs as an immune evasion mechanism (10), indicative of an interplay between Tregs and AML cells.

We demonstrated that target cell recognition by effector cells triggered the secretion of cytokines such as RANTES, MIP1α and MIP1β in our cytotoxicity assays and data published by others (36–38). Taking into account that previous studies showed alterations of NK cell functionality in myeloid malignancies (39) and limited clinical activity of ADCC competent antibodies against other targets (e.g. CD33, CD123) (40–42), investigating the cytotoxic potential of autologous patient derived NK cells would be of high interest. Since Tregs can inhibit NK cell activity through the release of immunosuppressive soluble factors such as TGF-β or IL-10 and direct cell-to-cell contact (12, 43), eliminating Tregs *via* ADCC-enhanced CD25 antibody treatment may trigger a positive feedback loop and lead to a boost in NK-cell functionality.

In our study, the FLT3-ITD mutant patients were enriched for CD25+ AML cells, which has also been described previously by others (5, 7). It is hypothesized that FLT3-ITD leads to the upregulation of *RET*, *IL2RA* and *CCL5* through Follistatin (44), providing some mechanistic basis for the strong association between FLT3-ITD and CD25 expression.

Furthermore, we observed that BCL-2 expression was higher on CD25+ AML clusters with an immature phenotype than on clusters with a mature phenotype. This finding is in line with prior studies that showed high BCL-2 expression in the LSC compartment (45, 46). This raises the question of a potential synergistic effect between CD25 Mab and the BCL-2 inhibitor venetoclax, especially since we found CD25+ AML clusters in all four patients treated with HMA-VEN and CD25 was more highly expressed on LSCs and immature AML cells. However, further investigations are required to understand if a combination strategy with HMA-VEN and CD25 Mab may prevent escape and subsequent relapse. On the other hand, CD25 Mab could potentially overcome resistance of BCL-2 low clusters to VEN treatment.

Therapeutic targeting of CD25 provides an opportunity for dual targeting of CD25+ AML cells and Tregs in a subset of AML patients. There are a few antibodies depleting CD25 expressing cells clinically available (47), and more are in preclinical development (48, 49). In our opinion, there are two major challenges in the development of CD25 depleting agents for cancer immunotherapy. Firstly, targeting CD25 should not interfere with IL-2 signaling for an optimal adaptive immunity formation. In AML blasts, IL-2 signaling did not induce proliferation (50) and high CD25 expression could likely be due to hyper activation of STAT5 (51), frequently reported for AML patients, potentially as a consequence of FLT-ITD mutations (52). It is tempting to speculate that in case of constitutive STAT5 signaling, further stimulation *via* IL-2 would not affect AML blast survival. Secondly, the drug should be able to spare beneficial CD25 positive cells (e.g. activated NK cells, activated tumor specific CD8 T cells) whilst eliminating unwanted CD25 positive cells (e.g. Treg, CD25+ AML blasts). ADCT-301 (camidanlumab tesirine) (53) is an antibody drug conjugate (ADC) composed of a human CD25-targeting monoclonal antibody conjugated to tesirine, a pyrrolobenzodiazepine (PBD) dimer cytotoxin. Camidanlumab tesirine has been tested in CD25-expressing hematological malignancies including CD25+ AML (NCT02588092, 34 patients in total) (54), relapsed/refractory (R/R) Hodgkin lymphoma (NCT04052997) and non-Hodgkin lymphoma (NCT02432235). In AML, the rapid clearance indicated that Q3W dosing (22/34 patients) may not be sufficient to reach a therapeutically active concentration. Subsequently, 2/9 patients treated with QW schedule to improve exposure achieved complete responses with incomplete hematologic recovery. However, the trial was terminated during dose escalation due to programmatic reasons (superior results in r/r Hodgkins lymphoma with an overall response of 71%). It is difficult to evaluate the potency of Camidanlumab tesirine in AML since the recommended dose was not determined. The encouraging findings warrant further evaluation of CD25 targeting compounds for the treatment of AML. In addition, the avidity binding component of RG6292 and ADCC/P as mechanism of action requires a certain target density to facilitate target cell killing. RG6292 allows for depletion of Tregs whilst activated CD8 T cells with low CD25 expression were able to expand (16). In the original description of Camidanlumab tesirine, it is postulated that cytotoxicity of the ADC drug is independent of CD25 target density (53). Therefore, this property may interfere with the formation of adaptive immunity by depletion of recently activated CD8 T cell clones and requires further evaluation.

Lastly, our proteomic analysis did not identify a unique LSC phenotype conserved across patients, which highlights the underlying heterogeneity in AML and plasticity in the LSC compartment (55, 56). However, immunotherapy is a relatively untapped therapeutic area in AML that alone or in combination with other targeted therapeutic modalities has the potential to eliminate the drug-resistant LSC population and offer cure.

The limitations of our study include the sample size and the source of primary material. Considering that frozen samples commonly have compromised innate cell activity, we needed to add allogeneic NK cells in the ADCC assay. Moreover, we tried to set up translatable mouse models to study the activity of CD25 Mab *in vivo* using syngeneic mice as well as stem cell humanized mice harboring AML tumor cell lines or AML PDx, respectively. Despite these attempts, we could not recapitulate a patient relevant mouse model to study the dual mode of action of CD25 Mab.

Hence, the identification of the patient population who may benefit most from CD25 Mab requires further characterization of the AML blast and microenvironment in a larger sample size, analyzing emerging clinical data from HMA-VEN trials, and testing *ex vivo* cell killing or the impact on effector T cell function on more treatment naïve and relapsed/refractory AML samples.

## Conclusions

Using high dimensional flow cytometry and computational analysis, we provided a deep characterization of AML patient samples and highlighted the heterogeneity in the pattern of expression of common AML targets currently under clinical investigation. We dissected the expression of CD25 on healthy T cells and malignant cells and highlighted that the blood offers a window into the bone marrow composition and could be used to ascertain Treg and CD25+ AML cells prevalence. Mode of action studies demonstrated that CD25 Mab depletes suppressive Tregs and has a direct cytotoxic effect on the CD25+ AML cells. CD25 targeting represents an attractive option for the treatment of AML, especially in patients where CD25 is expressed on LSC or immature AML cells. Considering the high relapse rate in AML, it would be important to understand if CD25 Mab could deepen responses and help prevent relapse. Our study warrants further exploration of CD25 Mab as combinatorial treatment with, for instance, FLT3 inhibitors or venetoclax.

## Data availability statement

The RNA sequencing datasets generated during the current study are available at the NCBI Sequence Read Archive (SRA) under the accession ID PRJNA855458 and PRJNA855476 for Study #1 and #2, respectively.

## Ethics statement

The studies involving human participants were reviewed and approved by the N.N Blokhin Russian Cancer Research Center RAMS Ethics Committee and the WCG Institutional Review Board. The patients/participants provided their written informed consent to participate in this study.

## Author contributions

LP designed the research study, performed the experiments, analyzed and interpreted data, wrote and reviewed the manuscript. KK interpreted the data, wrote and reviewed the manuscript. BM designed the research study, interpreted data and wrote the manuscript. MB performed the RNA-Sequencing analysis and gene expression, and wrote the manuscript. NK designed the RNA-Sequencing study, generated, curated and interpreted associated data, and wrote the manuscript. JE provided AML22 cells and input into interpretation of data. IH contributed to ADCC experiments. VG performed the RNA library preparation and sequencing. VK provided input into study design and interpretation of data. CK provided input into study design and interpretation of data. MA designed the research study, interpreted data and reviewed the manuscript. All authors contributed to the article and approved the submitted version.

## Glossary

**Table:**

ADC	Antibody drug conjugate
ADCC	Antibody-dependent cellular cytotoxicity
ADCP	Antibody-dependent cellular phagocytosis
Allo-SCT	Allogeneic Stem Cell Transplantation
AML	Acute Myeloid Leukemia
BMMC	Bone marrow mononuclear cell
CD	Cluster of differentiation
CLEC12A	C-type lectin domain family 12 member A
DC	Dendritic cell
Fc	Fragment crystallizable
FcR	Fc receptor
FMO	Fluorescence-minus-one
FoxP3	Forkhead Box P3
G-CSF	Granulocyte-colony stimulating factor
HD	Healthy donor
HLA	Human Leukocyte Antigen
HMA	DNA Hypomethylating Agent
HSC	Hematopoietic Stem cell
HSPC	Hematopoietic Stem and Progenitor cell
IFNγ	Interferon gamma
IgG1	Immunoglobulin G1
IL	Interleukin
IL2RA	IL-2 receptor alpha chain
IRB	Institutional Review Board
LAG3	Lymphocyte-activating 3
LSC	Leukemic Stem Cell
MCP-1	Monocyte chemoattractant protein-1
MFI	Median Fluorescence Intensity
MIP1α	Macrophage inflammatory protein-1 alpha
MIP1β	MIP1α, Macrophage inflammatory protein-1 beta
NK cell	Natural Killer cell
PBMC	Peripheral blood mononuclear cell
PC	Plasma cell
PD-1	Programmed cell death protein 1
pDC	Plasmacytoid dendritic cell
RANTES	Regulated upon Activation, Normal T cell Expressed and secreted
RNAseq	RNA sequencing
SEM	Standard error of the mean
TIGIT	T cell immunoreceptor with Ig and ITIM domain
TIM-3	T cell immunoglobulin and mucin-domain containing-3
TNFα	Tumor necrosis factor alpha
Treg	Regulatory T cell
UMAP	Uniform Manifold Approximation and Projection
VEGF	Vascular endothelial growth factor
VEN	Venetoclax
WT1	Wilms' tumor protein
