# Supplementary material for: CD25 targeting with the afucosylated human IgG1 antibody RG6292 eliminates regulatory T cells and CD25+ blasts in acute myeloid leukemia
Source: Front Oncol. 2023 May 2;13:1150149. doi: 10.3389/fonc.2023.1150149 (PMC10185852; doi:10.3389/fonc.2023.1150149)

Table S1: Metadata patient samples analyzed in Study #1

Patient ID	Sample ID	Supplier	Sample type	Date of collection	Sex	Age	Clinical diagnosis	FLT3-ITD status	Disease status	Treatment category
P01	P01	DLS	BMMC	9/4/2019	Male	69	AML	positive	Newly diagnosed	untreated
P02	P02	DLS	BMMC	6/25/2019	Male	47	AML	negative	Newly diagnosed	untreated
P03	P03	DLS	BMMC	8/7/2019	Female	62	AML	negative	Newly diagnosed	untreated
P04	P04	DLS	BMMC	6/3/2019	Female	67	AML	negative	Newly diagnosed	untreated
P05	P05	DLS	BMMC	12/3/2019	Male	63	AML	negative	Newly diagnosed	untreated
P06	P06	DLS	BMMC	11/7/2019	Female	74	AML	negative	Newly diagnosed	untreated
P07	P07	DLS	BMMC	1/2/2020	Female	78	AML	positive	Newly diagnosed	untreated
P08	P08	DLS	BMMC	1/31/2020	Male	36	AML	negative	Newly diagnosed	untreated
P09	P09	DLS	BMMC	8/2/2019	Male	64	AML	positive	Newly diagnosed	untreated
P10	P10	DLS	BMMC	8/5/2019	Male	63	AML	negative	Newly diagnosed	untreated
P11	P11	DLS	BMMC	10/8/2019	Male	30	AML, M5	negative	Newly diagnosed	untreated
P12	P12	DLS	BMMC	4/29/2020	Female	76	AML	negative	Newly diagnosed	untreated
P13	P13	DLS	BMMC	1/16/2020	Male	69	AML	negative	Newly diagnosed	untreated
P14	P14	DLS	BMMC	12/16/2019	Male	63	AML	negative	Newly diagnosed	untreated

Table S2: Metadata patient samples analyzed in Study #2

Patient ID	Sample ID	Supplier	Sample type	Date of collection	Sex	Age	Clinical diagnosis	FLT3-ITD status	Disease status	Treatment category
P01	S01	DLS	PBMC	12.12.2020	Female	49	AML, M5	positive	Newly diagnosed	untreated
P01	S02	DLS	BMMC	12.12.2020	Female	49	AML, M5	positive	Newly diagnosed	untreated
P02	S03	DLS	BMMC	19.11.2020	Male	56	AML	positive	Newly diagnosed	untreated
P02	S04	DLS	PBMC	19.11.2020	Male	56	AML	positive	Newly diagnosed	untreated
P02	S05	DLS	BMMC	17.12.2020	Male	56	AML	positive	Relapse/ refractory	chemo
P02	S06	DLS	PBMC	17.12.2020	Male	56	AML	positive	Relapse/ refractory	chemo
P03	S07	DLS	PBMC	01.12.2020	Female	53	AML	positive		chemo
P03	S08	DLS	BMMC	17.12.2020	Female	53	AML	positive	Relapse/ refractory	chemo
P04	S09	DLS	PBMC	07.12.2020	Male	56	AML, M4	positive	Relapse/ refractory	chemo
P04	S10	DLS	BMMC	07.12.2020	Male	56	AML, M4	positive	Relapse/ refractory	chemo
P05	S11	DLS	PBMC	14.01.2021	Female	56	AML, M4	positive	Newly diagnosed	untreated
P05	S12	DLS	BMMC	14.01.2021	Female	56	AML, M4	positive	Newly diagnosed	untreated
P06	S18	DLS	PBMC	05.11.2020	Male	73	AML	positive	Stable disease	VEN-HMA
P06	S19	DLS	BMMC	05.11.2020	Male	73	AML	unknown	Stable disease	VEN-HMA
P07	S20	DLS	PBMC	24.02.2020	Female	62	AML	positive	Relapse/ refractory	Chemo + HMA
P07	S21	DLS	BMMC	24.02.2020	Female	62	AML	positive	Relapse/ refractory	Chemo + HMA
P08	S22	DLS	PBMC	21.04.2020	Male	71	AML, M5	negative	Relapse/ refractory	HMA
P08	S23	DLS	BMMC	21.04.2020	Male	71	AML, M5	negative	Relapse/ refractory	HMA
P09	S24	DLS	BMMC	11.06.2020	Male	72	AML	negative	Newly diagnosed	Untreated
P09	S25	DLS	PBMC	11.06.2020	Male	72	AML	unknown	Newly diagnosed	Untreated
P09	S26	DLS	BMMC	13.08.2020	Male	72	AML	negative	Relapse/ refractory	Chemo
P09	S27	DLS	PBMC	12.08.2020	Male	72	AML	unknown	Relapse/ refractory	Chemo
P10	S28	DLS	BMMC	27.10.2020	Female	71	AML	negative	Relapse/ refractory	HMA
P11	S29	DLS	BMMC	16.12.2019	Male	42	AML	negative	Stable	HMA
P12	S30	DLS	BMMC	22.01.2020	Male	76	AML	negative	Relapse/ refractory	HMA
P13	S31	DLS	PBMC	13.04.2020	Female	63	AML	negative	Relapse/ refractory	HMA
P14	S32	DLS	PBMC	26.08.2019	Female	83	AML	negative	Stable disease	HMA
P15	S33	DLS	PBMC	10.01.2019	Female	46	AML	positive	Stable disease	HMA-VEN

P16	S34	DLS	PBMC	04.05.2020	Female	61	AML	unknown	Stable disease	HMA-VEN
P16	S35	DLS	PBMC	03.02.2020	Female	61	AML	negative	Unknown	HMA-VEN
P17	S36	DLS	PBMC	20.07.2020	Female	82	AML	negative	Stable disease	HMA-VEN
P18	S37	DLS	BMMC	26.08.2020	Male	63	AML	positive	Relapse/ refractory	HMA
P10	S38	DLS	BMMC	11.06.2020	Female	71	AML	negative	Newly diagnosed	Untreated
P10	S39	DLS	PBMC	11.06.2020	Female	71	AML	unknown	Newly diagnosed	Untreated
P19	S40	ProteoGenex	PBMC	13.09.2019	Female	61	AML	negative	Relapse/ refractory	HMA
P20	S41	ProteoGenex	PBMC	03.11.2020	Female	56	AML	negative	Relapse/ refractory	HMA
P21	S42	ProteoGenex	BMMC	23.11.2020	Female	71	AML, M2	negative	Newly diagnosed	Untreated
P21	S43	ProteoGenex	PBMC	23.11.2020	Female	71	AML, M2	negative	Newly diagnosed	Untreated
P22	S44	ProteoGenex	PBMC	23.01.2019	Male	74	AML, M2	negative	Newly diagnosed	Untreated
P23	S45	ProteoGenex	BMMC	19.02.2019	Female	85	AML, M2	negative	Newly diagnosed	Untreated
P23	S46	ProteoGenex	PBMC	19.02.2019	Female	85	AML, M2	negative	Newly diagnosed	Untreated

Table S3: Flow cytometry panel for the characterization of patient samples in study #1

Reagent	Supplier	Cat. No.	Clone
BV570-P anti-human HLA-DR	BD Biosciences	Custom-made	G46-6
BB630-P anti-human PD-L1	BD Biosciences	Custom-made	MIH1
BB790-P anti-human CD117	BD Biosciences	Custom-made	YB5.B8
APC-R700 anti-human CD33	BD Biosciences	659122	P67.6
PE-CF594 anti-human CD206	BD Biosciences	564063	19.2
BUV395 anti-human CD45RA	BD Biosciences	740298	HI100
BUV615 anti-human Lag3	BD Biosciences	752362	T47-530
BV605 anti-human CD56	Biolegend	362537	5.1H11
PE-Cy7 anti-human CD127	Biolegend	351320	A019D5
BV421 anti-human Ki67*	Biolegend	350506	Ki67
A647 anti-human FoxP3*	Biolegend	320214	259D
PE Anti-human CD25	R&D Systems	FAP1020P-100	24212
BUV496 anti-human CD8a	BD Biosciences	612943	RPA-T8
BUV805 anti-human CD45	BD Biosciences	612892	HI30
BV711 anti-human TIGIT	BD Biosciences	747839	741182
BB515 anti-human CD38	BD Biosciences	564499	HIT2
BB700 anti-human TIM3	BD Biosciences	746178	7D3
APC-Cy7 anti-human CD4	Biolegend	357416	A161A1
BV480 anti-human CD34	BD Biosciences	746688	8G12
BV750 anti-human CD47	BD Biosciences	747165	B6H12
PE-Cy5 anti-human CD123	BD Biosciences	561009	9F5
BUV563 anti-human CD14	BD Biosciences	741441	MϕP9
BUV661 anti-human CD16	BD Biosciences	750284	3G8
BV650 anti-human CD32	BD Biosciences	740572	FLI8.26
BUV737 anti-human PD1	BD Biosciences	612792	EH12.1
BV785 anti-human CD3	Biolegend	300474	UCHT1
Fixable Viability Stain 440UV	BD Biosciences	566332	
Brilliant Stain Buffer Plus	BD Biosciences	566385	

P: Prototype
* Intra-cellular antigens

Table S4: Flow cytometry panel for the characterization of patient samples in study #2

Reagent	Supplier	Cat. No.	Clone
BUV395 anti-human CD45RA	BD Biosciences	740298	HI100
Unconjugated rabbit anti-human WT-1*	Cell Signaling Technologies	83535S	D8I7F
AF350 anti-rabbit IgG	Thermo Fisher	A-11046	Polyclonal
BUV496 anti-human CD8a	BD Biosciences	612943	RPA-T8
BUV563 anti-human CD11c	BD Biosciences	741358	B-ly6
BUV615 anti-human CD71	BD Biosciences	751461	M-A712
BUV661 anti-human CD16	BD Biosciences	750284	3G8
BUV737 anti-human PD1	BD Biosciences	612792	EH12.1
BUV805 anti-human CD45	BD Biosciences	612892	HI30
BV421 anti-human Ki67*	Biolegend	350506	Ki67
cFluor 450 anti-human CD14	Cytek	SKU R7-20004	M5E2
BV480 anti-human CD34	BD Biosciences	746688	8G12
BV510 anti-HLA-A2	BD Biosciences	740184	BB7.2
BV570-P anti-human HLA-DR	BD Biosciences	Custom-made	G46-6
BV605 anti-human CD56	Biolegend	362537	5.1H11
BV650 anti-human CD32	BD Biosciences	740572	FLI8.26
BV711 anti-human TIGIT	BD Biosciences	747839	741182
BV750 anti-human CD47	BD Biosciences	747165	B6H12
BV785 anti-human CD3	Biolegend	300474	UCHT1
BB515 anti-human CD38	BD Biosciences	564499	HIT2
KIRAVIA Blue 520 anti-human CD235A	Biolegend	Custom-made	HIR2
Spark Blue 550 anti-human CD4	Biolegend	344656	SK3
BB630-P anti-human CLEC12A	BD Biosciences	Custom-made	50C1
BB630-P anti-human CD28	BD Biosciences	Custom-made	CD28.2
Biotinylated anti-human LAG3	Biolegend	369327	11C3C65
PerCP Streptavidin	Biolegend	405213	
BB700 anti-human TIM3	BD Biosciences	746178	7D3
PerCP-efluor710 anti-human CD1c	Thermo Fisher	46-0015-42	L161
BB755-P anti-human 4-1BB	BD Biosciences	Custom-made	4B4-1
BB790-P anti-human CD117	BD Biosciences	Custom-made	YB5.B8
PE Anti-human CD25	R&D Systems	FAP1020P-100	24212
PE-CF594 anti-human Bcl-2*	BD Biosciences	563601	Bcl-2/100
PE-Cy5 anti-human CD123	BD Biosciences	561009	9F5
PE/Fire 700 anti-human CD141	Biolegend	Custom-made	M80
PE-Cy7 anti-human CD127	Biolegend	351320	A019D5
APC anti-human CD41	Biolegend	303710	HIP8
A647 anti-human FoxP3*	Biolegend	320214	259D
Spark NIR 685 anti-human CD69	Biolegend	310957	FN50
APC-R700 anti-human CD33	BD Biosciences	659122	P67.6
APC-Fire 750 anti-human CD206	Biolegend	321134	15-2
APC/Fire 810 anti-human CD19	Biolegend	302272	HIB19
Zombie NIR viability dye	Biolegend	423106	

Figure S1: Analysis workflow of flow cytometry data

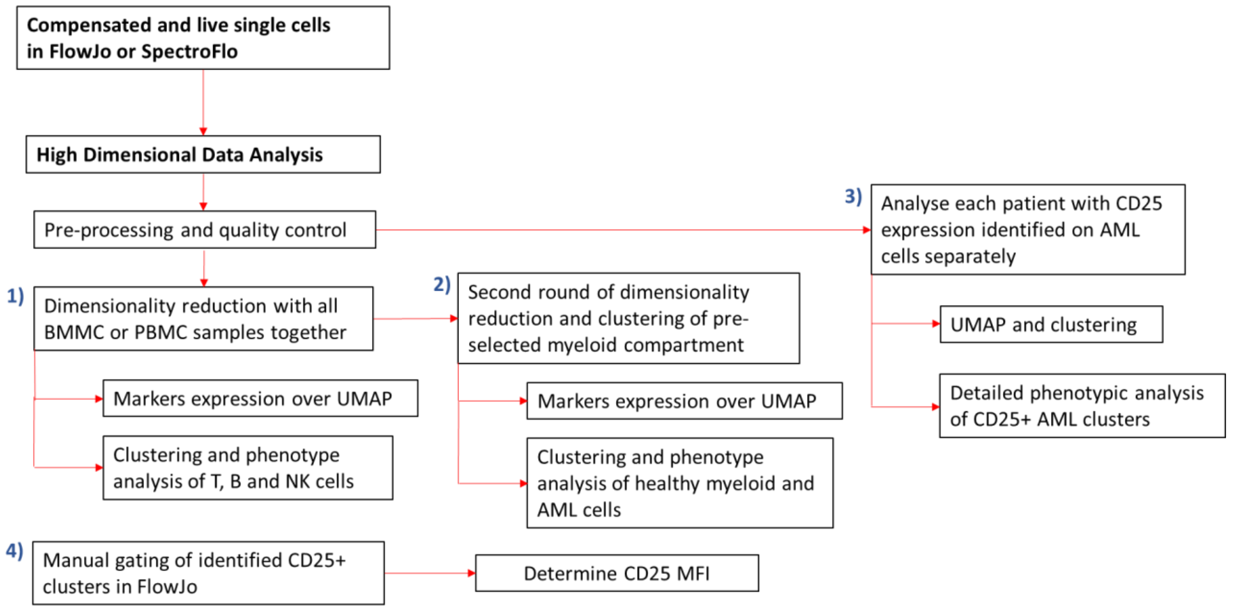

Figure S2: Representative example of the gating strategy to identify CD25+ cell populations

(A) Gating strategy to study CD25 expression in BM lymphoid populations presented in Figure 2.
(B) Gating strategy to study CD25 expression in AML cells presented in Figure 5A. The set of markers used to identify CD25+ AML cells was unique to each patient and based on the phenotype of CD25+ AML clusters identified via computational analysis and shown in Figure 4A. Co-expression of CD34 and CD38 within the CD25+ AML population is also exemplified in the gating strategy and shown in Figure 4C.

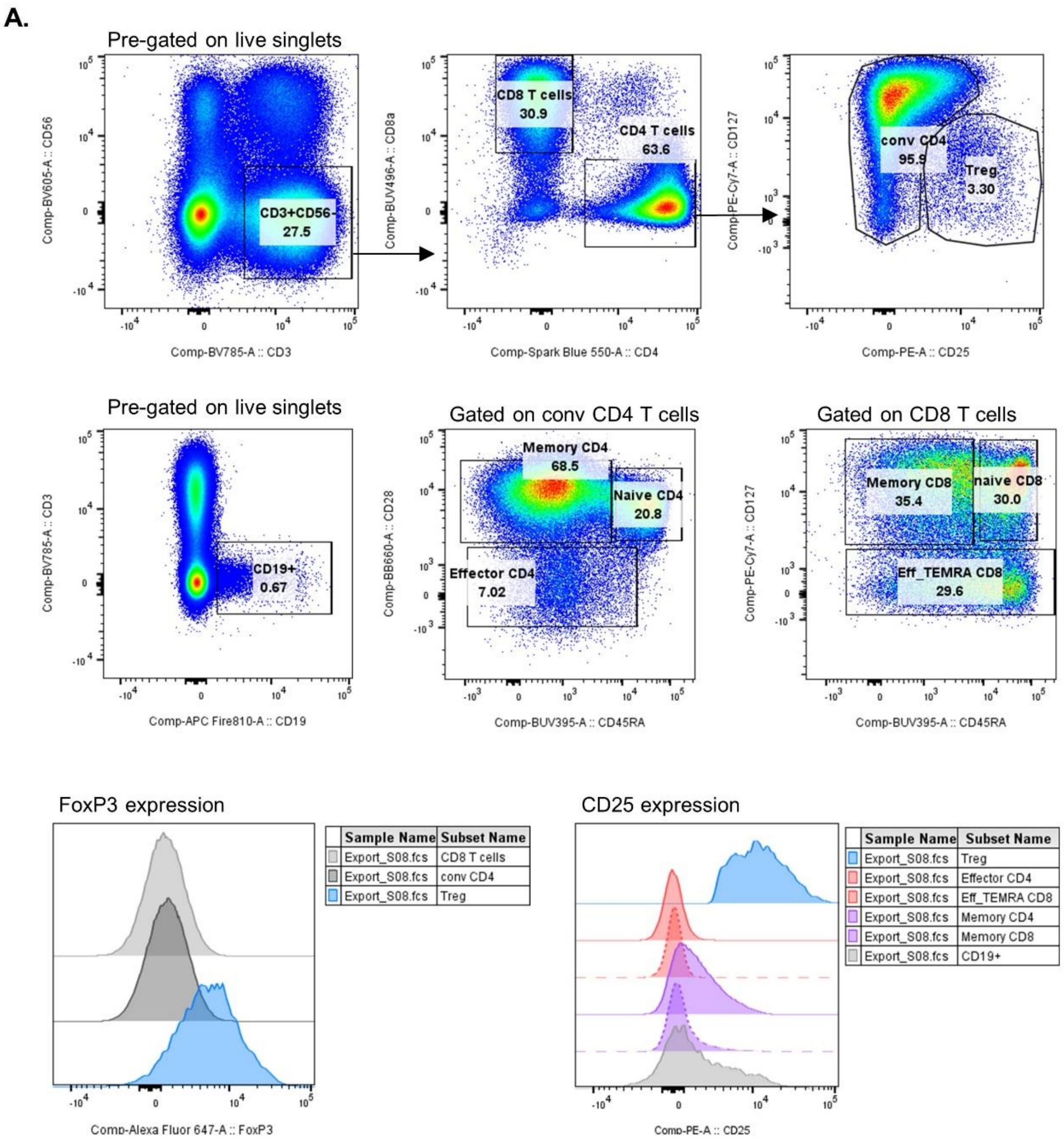

B.

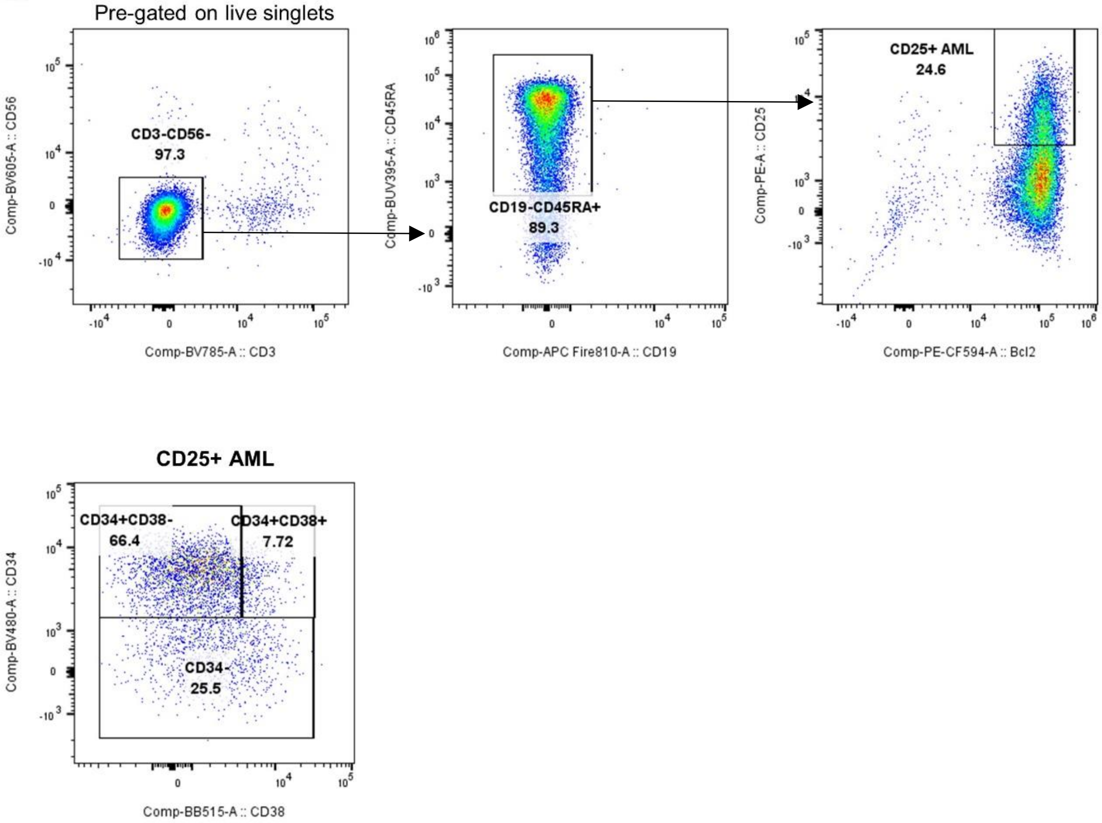
Pre-gated on live singlets
CD3-CD56-
97.3
Comp-BV605-A :: CD56
Comp-BV785-A :: CD3
CD19-CD45RA+
89.3
Comp-BUV395-A :: CD45RA
Comp-APC Fire810-A :: CD19
CD25+ AML
24.6
Comp-PE-A :: CD25
Comp-PE-CF594-A :: Bcl2
CD25+ AML
CD34+CD38-
66.4
CD34+CD38+
7.72
CD34-
25.5
Comp-BV480-A :: CD34
Comp-BB515-A :: CD38

Figure S3: AML clusters heterogeneity

(A) Patient ID overlaid on UMAP embedding of all BMMC samples analyzed in Study #1. Dotted line represents the border of the myeloid compartment. Color is specific to each patient and highlights that some AML cells have a unique phenotype that is not shared across patients.
(B) Myeloid clusters overlaid on UMAP embedding of all BMMC samples analyzed in Study #2. See Methods for details.
(C) Table summarizing the prevalence of common AML antigens shown as proportion of patients in which a given marker was identified on AML cells as well as the maximum frequency of AML cells positive for each marker.
(D) Dot plot depicting the expression of 30 markers on annotated AML clusters identified in the bone marrow of samples analyzed in Study #2. Color code indicates the median expression (logicle transformed MFI); point size indicates the fraction of cells positive for each marker.

A.

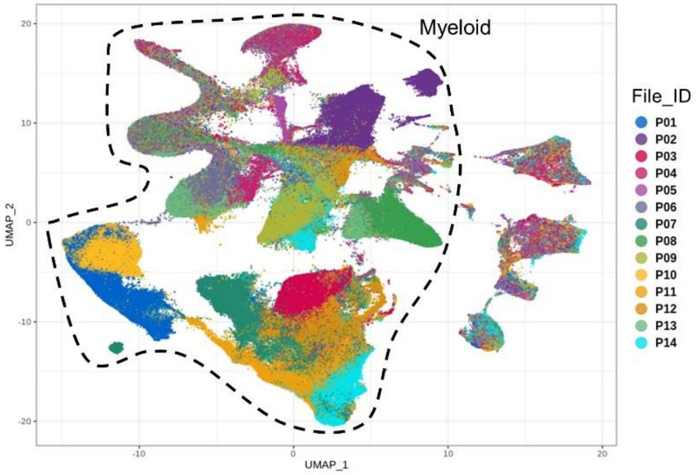

B.

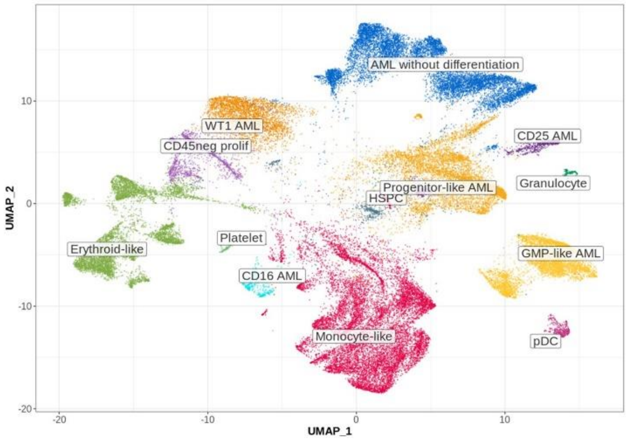

C.

Marker	Marker+ patients (%)	Marker+ AML cells (% of myeloid)
CD25	~ 45%	Up to 30%
WT-1	~ 50%	Up to 85%
CD117	~ 65%	Up to 75%
CD123	~ 75%	Up to 75%
CD33	~ 90%	Up to 95%
CD34	~ 60%	Up to 80%
CD14	~ 45%	Up to 80%
CD206	~ 50%	Up to 65%
CD38	~ 85%	Up to 80%
CLEC12A	~ 65 %	Up to 95%
TIM-3	~ 75 %	Up to 80%
CD47	100%	Up to 98%

D.

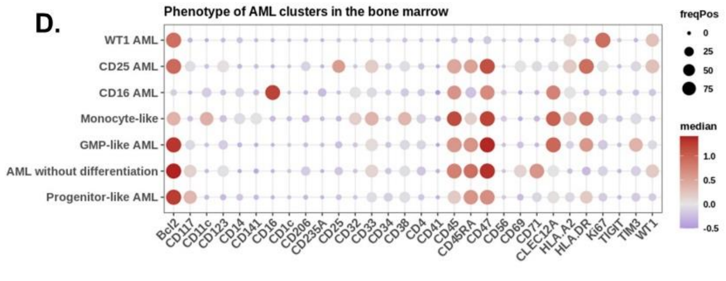

Figure S4: Phenotypes of CD25+ AML clusters

Summary of findings for the 14 patients analyzed individually and whose phenotypes are represented in Figure 4A. Immature and mature phenotypes refer to clusters with or without CD34 expression, respectively.

	Patient ID	Disease status	Treatment category	CD25+ cluster (% of AML)	Phenotype (of CD25+ AML)	Comment
Study #1	P07	Newly Diagnosed	None	30 %	Immature & Mature	Only CD34 expressing AML cluster.
Study #1	P10	Newly Diagnosed	None	2.9 %	Immature	Most AML cells express CD34.
Study #1	P01	Newly Diagnosed	None	1.6 %	Immature	Fraction of AML cells are CD34+CD25-.
Study #1	P06	Newly Diagnosed	None	0.6 %	Immature	Fraction of AML cells are CD34+CD25-.
Study #2	P02	Newly Diagnosed & refractory to Chemo	None & Chemo	0.1-2.4 %	Immature	Only CD34 expressing AML cluster.
Study #2	P09	Newly Diagnosed & refractory to Chemo	None & Chemo	30 % in BM at diagnosis Not detected in PB	Mature & Immature	Fraction of AML cells are CD34+CD25-.
Study #2	P05	Newly diagnosed	None	14.1-17.8 %	Mature	No CD34 expressing cells. 0.2% healthy HSC in BM
Study #2	P17	Stable disease	HMA-VEN	9.8 %	Mature	No CD34 expressing cells
Study #2	P15	Stable disease	HMA-VEN	26 %	Immature	Most AML cells express CD34
Study #2	P01	Newly diagnosed	None	17-20 %	Immature	Only CD34 expressing AML cluster.
Study #2	P07	Relapse/refractory	HMA	4.6-5.8 %	Mature	1% of AML cells express CD34
Study #2	P08	Relapse/refractory	HMA	11.7 %	Immature	Only CD34 expressing AML cluster. 0.3% healthy HSC in BM
Study #2	P16	Stable disease	HMA-VEN	5-43 %	Immature	5% on 1st collection date, 43% 3 months later
Study #2	P06	Stable disease	HMA-VEN	0.5-5.8%	Mature & Immature	Only CD34 expressing AML cluster. 0.4% healthy HSC in BM

Figure S5: Prevalence and expression of CD25+ AML cells according to prior treatment

(A) Abundance of CD25+ AML cells in PBMC or BMMC samples according to the patients' treatment category (treatment naïve, treated with chemotherapy, HMA or HMA-VEN). Data compiled from Study #1 and #2. One-way ANOVA, Tukey's multiple comparison test. The proportion of patients for which CD25 expression was detected on AML cells in each category is shown below the bar graph.
(B) CD25 expression (MFI) on CD25+ AML cells in Study #2, according to the patients' treatment category (untreated, treated with chemotherapy and/or HMA, HMA-VEN).

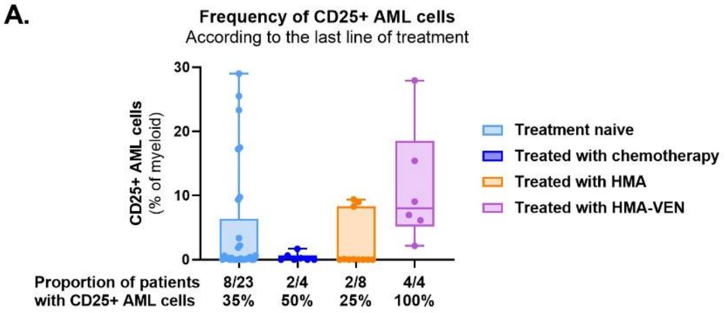

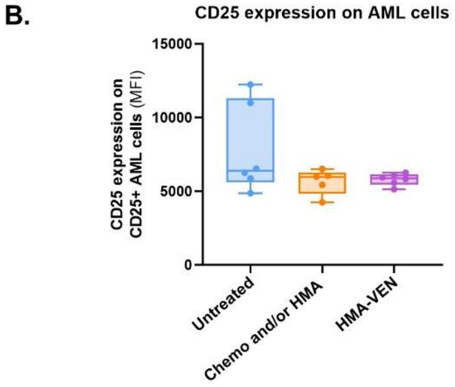

Supplement: Supplementary file 1 [file Presentation_1.pdf]
